# Efficient Multiplex Gene Repression by CRISPR-dCpf1 in *Corynebacterium glutamicum*

**DOI:** 10.3389/fbioe.2020.00357

**Published:** 2020-04-24

**Authors:** Mingyue Li, Jiuzhou Chen, Yu Wang, Jiao Liu, Jingwen Huang, Ning Chen, Ping Zheng, Jibin Sun

**Affiliations:** ^1^College of Biotechnology, Tianjin University of Science and Technology, Tianjin, China; ^2^Key Laboratory of Systems Microbial Biotechnology, Tianjin Institute of Industrial Biotechnology, Chinese Academy of Sciences, Tianjin, China; ^3^School of Biology and Biological Engineering, South China University of Technology, Guangzhou, China

**Keywords:** *Corynebacterium glutamicum*, CRISPR-dCpf1, multiplex gene repression, metabolic engineering, lysine

## Abstract

*Corynebacterium glutamicum* is an important workhorse for industrial production of diversiform bioproducts. Multiplex control of metabolic pathway genes is crucial for maximizing biosynthesis of desired products. However, few tools for simultaneously regulating multiple genes in *C. glutamicum* have been reported. Here, a CRISPR-dCpf1-based multiplex gene repression system was developed for *C. glutamicum*. This system successfully repressed two fluorescent reporter genes simultaneously by expressing a dCpf1 (E1006A, D917A) and a designed single crRNA array. To demonstrate applications of this CRISPR-dCpf1 system in metabolic engineering, we applied this system to repress four genes involved in lysine biosynthesis (*gltA*, *pck*, *pgi*, and *hom*) with a single array, which increased the lysine titer and yield for over 4.0-fold. Quantitative PCR demonstrated that transcription of all the four endogenous target genes were repressed by over 90%. Thus, the CRISPR-dCpf1 system is a simple and effective technique for multiplex gene repression in *C. glutamicum* and holds promise for metabolic engineering of *C. glutamicum* to produce valuable chemicals and fuels.

## Introduction

*Corynebacterium glutamicum*, a non-pathogenic Gram-positive bacterium, is an important platform strain and has been widely used for industrial production of various amino acids, biochemicals, and biofuels (Becker and Wittmann, 2012; Yokota and Ikeda, 2017). To maximize biosynthesis of desired product, expression of multiple metabolic pathway genes needs to be balanced (Lee et al., 2019). However, identification of the optimum expression level for each target gene is time-consuming. Considering the underlying intricacies and interrelationships of metabolic pathways, optimizing the expression of target genes one by one usually cannot lead to desired outcomes (Schafer et al., 1994; Ikeda and Nakagawa, 2003; Okibe et al., 2011). Therefore, development of efficient multiplex gene regulation techniques is in urgent demand.

Recently, synthetic small regulatory RNA (sRNA) system and class 2 type II-A clustered regularly interspaced short palindromic repeats (CRISPR)/CRISPR-associated protein (Cas) system (Cas9) have been repurposed as RNA interference (RNAi) and CRISPR interference (CRISPRi) tools, respectively, for gene repression in *C. glutamicum* (Cleto et al., 2016; Park et al., 2019; Sun et al., 2019). However, for both sRNA-mediated RNAi and deactivated Cas9 (dCas9)-mediated CRISPRi, a sRNA or CRISPR RNA (crRNA) will be required for each target gene repression because these systems are incapable of processing RNA arrays into individual functional RNAs. By recruiting native RNase III and expressing a dCas9:crRNA:transactivating crRNA (tracrRNA) complex, a crRNA array processing system was developed for dCas9-mediated multiplex transcriptional repression in *Escherichia coli* (Cress et al., 2015). However, such system has not been established in *C. glutamicum* due to limited knowledge of functional RNase. As a result, multiple RNA expression cassettes with high sequence and architecture similarity need to be constructed for multiplex gene regulation in *C. glutamicum*, which is difficult to assemble *in vitro* and also unstable *in vivo* (Reis et al., 2019).

Unlike Cas9, class 2 type V-A CRISPR effector Cpf1 (also known as Cas12a) possesses both DNase and RNase activities, which can process crRNA arrays into mature crRNAs and offer an alternative tool for genetic modifications (Luo et al., 2016). Furthermore, the direct repeat sequence of Cpf1 crRNA (∼20 nt) is much shorter than the handle sequence of Cas9 crRNA (∼60 nt) (Fonfara et al., 2016; Liu et al., 2017), which makes synthesis and assembly of Cpf1 crRNA array cheaper and easier. These properties provide distinct advantages to perform multiplex gene editing and perturbation (Zetsche et al., 2015). Over the last 2 years, CRISPR-dCpf1 (DNase-deactivated Cpf1) systems have been employed for gene repression in several microorganisms, such as *E. coli*, *Streptomyces*, and *Yarrowia lipolytica* (Kim et al., 2017; Zhang et al., 2017; Li et al., 2018; Zhang et al., 2018). Until very recently, Liu and colleagues reported the first attempt to use catalytically active Cpf1 with truncated crRNAs for gene repression in *C. glutamicum* (Liu et al., 2019). However, the low repression efficiency (up to 60%) and high risk of introducing double-stranded DNA breaks (DSBs) would limit its application. CRISPR-dCpf1 system that is capable of processing crRNA arrays but will not generate DSBs has not been systematically explored in *C. glutamicum* so far.

In this study, CRISPR-dCpf1 from *Francisella novicida* was employed for gene repression and metabolic engineering of *C. glutamicum*. By optimizing dCpf1 expression and testing different dCpf1 variants, an efficient CRISPRi system was successfully established in *C. glutamicum*, which simultaneously repressed expression of multiple target genes with high efficiencies over 90%. A Golden Gate assembly-based method was also developed for simple and rapid assembly of crRNA array. To demonstrate an application of this CRISPR-dCpf1 system in metabolic engineering of *C. glutamicum*, combinational repression of four potential target genes was conducted to maximize lysine production. The CRISPR-dCpf1-mediated multiplex gene repression technique developed here will enable the rapid development of high-performance *C. glutamicum* strains.

## Materials and Methods

### Bacterial Strains and Cultivation Conditions

Bacterial strains used in this study are listed in Supplementary Table S1. *E. coli* DH5α and DB.3.1 were used for the plasmid construction and cultivated in Luria-Bertani (LB) medium (5 g/L yeast extract, 10 g/L tryptone, 10 g/L NaCl) at 37°C. Kanamycin (50 μg/mL) or chloramphenicol (20 μg/mL) was added to LB medium as required. *C. glutamicum* strains were cultivated at 30°C in LB medium supplement with 5 g/L glucose (LBG medium). Kanamycin (25 μg/mL), chloramphenicol (5 μg/mL), or isopropyl-β-D-thiogalactopyranoside (IPTG) (1 mM) was added when necessary.

### Plasmid Construction

All the plasmids and primers used in this study are listed in Supplementary Tables S1, S2, respectively. *E. coli–C. glutamicum* shuttle expression vector pXMJ19 was used to express *dCpf1.* The E1006A mutation of Cpf1 from *F. novicida* was first introduced by PCR using primer pair pY003-E1006A-F/R. Then the *dCpf1* gene was amplified using the primer pair pXM-01-F/R and cloned into the *Hin*dIII and *Bam*HI site of pXMJ19, generating plasmid pXM-01. To optimize the expression of *dCpf1*, the start codon (ATG) of *dCpf1* was first replaced by GTG with primer pair pXM-02-GTG-F/R, resulting in plasmid pXM-02. Then the original ribosome binding site (RBS) of *dCpf1* in pXM-01 and pXM-02 (RBS1) was replaced with RBS2 and RBS3 (Supplementary Table S3) chosen from previously constructed libraries (Zhang et al., 2015) by PCR, resulting in plasmids pXM-03, pXM-04, pXM-05, and pXM-06, respectively. To replace the E1006A mutation in dCpf1 with D917A mutation, D917A mutation was first introduced into pXM-04 by PCR using primer pair pXM-07-F/R, generating plasmid pXM-07. Then the E1006A mutation of dCpf1 in pXM-07 was reversed by PCR using primer pair pXM-08-F/R, generating plasmid pXM-08.

*E. coli–C. glutamicum* shuttle expression vector pEC-XK99E was used to express crRNA. To construct a basic plasmid, the initial P*_*trc*_* promoter was replaced with a constitutive promoter (P*_11__*F*_*) by PCR using primer pair pEC-01-11F-F/R. Then the backbone of pEC-01 was amplified with primer pair and pEcrRNA-F/R. A *ccdB* cassette was amplified from pgRNA-*ccdB* (Wang et al., 2018b) with primer pair pEC-02-*ccdB*-F/R. These two PCR fragments were purified and ligated to generate plasmid pEC-02. Protospacers possessing the requisite 5′ PAM sequence (BTTV) were identified near the start codon of the coding region, and 23 nucleotides downstream of the PAM were selected as the spacer. Two oligonucleotides were annealed and assembled into *Bbs*I-digested pEC-02 backbone using a Golden Gate assembly method. All single-stranded DNA (ssDNA) oligonucleotides utilized for construction of crRNAs were listed in Supplementary Table S4.

### Dual-Fluorescence Reporter Strain Construction

To integrate a *gfp* expression cassette downstream *lysA* gene of the ATCC 13032::*rfp* chromosome and construct a dual-fluorescence reporter strain, plasmid pK18*mobsacB-gfp* was first constructed. To this end, *gfp* gene was amplified from plasmid pXM*-gfp* (Sun et al., 2019) using primer pair *gfp*-F/R. Then, the plasmid backbone of pK18*mobsacB* (Schafer et al., 1994), upstream and downstream recombination arms (about 1,000 bp) targeting the *lysA* locus were amplified with primer pairs pK18*mobsacB*-F/R, *gfp*-up-F/R and *gfp*-down-F/R, respectively. Finally, these PCR fragments were ligated using the CloneExpress MultiS One Step Cloning Kit (Vazyme Biotech, Nanjing, China). pK18*mobsacB-gfp* was transformed into strain ATCC 13032::*rfp* and markerless *gfp* expression cassette insertion was performed as described previously (Xu et al., 2014).

### Fluorescence Intensity Determination

The overnight cultures of *C. glutamicum* were transferred to fresh LBG medium supplemented with 1 mM IPTG to induce dCpf1 expression. After cultivated at 30°C and with shaking at 220 rpm for 24 h, cells were harvested by centrifugation at 5,000 × *g* for 10 min, washed once, and re-suspended in phosphate buffer (pH 7.4). Red fluorescent protein (RFP) and green fluorescent protein (GFP) fluorescence intensities were determined using a microplate reader (SpectraMax M5, Molecular Devices, RFP: λ excitation = 560 nm, λ emission = 607 nm; GFP: λ excitation = 488 nm, λ emission = 520 nm). The fluorescence intensities were normalized with OD_600_.

### Determination of Relative Transcriptional Level

For RNA extraction, cells were collected after 24 h cultivation. Total RNA extraction, reverse transcription and quantitative PCR (qPCR) were performed according to protocols described previously (Wang et al., 2018a). Briefly, RNA was extracted from the cell pellet using an RNAprep Pure Cell/Bacteria kit (Tiangen Biotech, Beijing, China). Then, cDNA was synthesized using random primers and a FastQuant RT kit (Tiangen Biotech, Beijing, China). The resultant cDNA was used as a template for qPCR analysis. The total RNA sample was used as a template for amplifying the target gene by PCR and no product could be detected by electrophoresis, suggesting that genomic DNA contamination during RNA extraction was minimal. Specific primers for qPCR were designed with Beacon Designer software v7.7 (PREMIER Biosoft International, United States). qPCR was performed by using a SuperReal Premix SYBR green kit (Tiangen Biotech, Beijing, China) and the Applied Biosystems 7,500 real-time PCR system (Thermo Fisher Scientific, United States) according to the manufacturer’s instructions. The gene encoding 16s rRNA was used as a reference for signal normalization. Data analysis was performed according to procedures described previously (Schmittgen and Livak, 2008).

### Lysine Production and Analytical Methods

*C. glutamicum* SCgL30 with a feedback deregulated aspartokinase (T311I) was used for lysine production. Strain SCgL30 and its derivatives were cultivated in LBG medium at 30°C and with shaking at 220 rpm. The overnight cultures were transferred into 24-well plates with 600 μL fermentation medium (80 g/L glucose, 8 g/L yeast extract, 9 g/L urea, 1.5 g/L K_2_HPO_4_⋅3H_2_O, 0.01 g/L MnSO_4_, 0.6 g/L MgSO_4_⋅7H_2_O, 0.01 g/L FeSO_4_⋅7H_2_O, 42 g/L MOPS) to an optical density at 600 nm (OD_600_) of 0.5. Then, cells were cultivated for 24 h at 30°C and with shaking at 800 rpm in INFORS Microtron (INFORS HT Multitron Pro, Switzerland). Samples were taken periodically and glucose and lysine concentrations were quantified using an SBA-40D biosensor analyzer (Institute of Biology of Shandong Province Academy of Sciences, Shandong, China). Cell biomass was determined as OD_600_ with a UV-1800 spectrophotometer (Shimadzu, Kyoto, Japan) after proper dilution with distilled water.

## Results

### Development of a CRISPR-dCpf1-Based Gene Repression System for *C. glutamicum*

**FIGURE 1:**
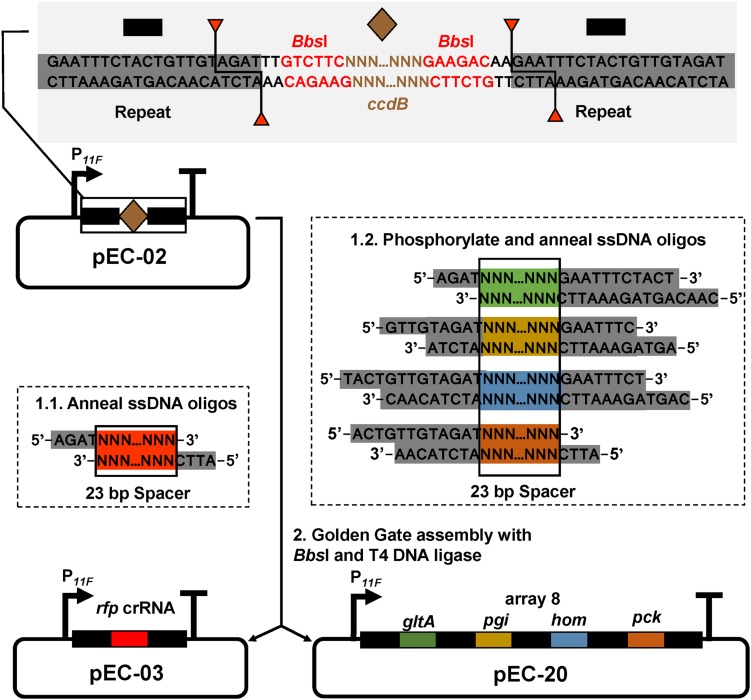
crRNA assembly strategy. pEC-02 harbors a constitutive promoter (P*_11__*F*_*), two DRs (black rectangle), and a *ccdB* cassette (brown diamond) flanked by two *Bbs*I sites (red font). Each 43 bp spacer-DR brick is assembled by 5′ phosphorylation and annealing of two offset complementary ssDNA oligonucleotides. For crRNA with a single targeting spacer, the ends of ssDNA oligonucleotides were designed as sequences complementary to the sticky ends of *Bbs*I in plasmid pEC-02. For crRNA arrays, the ends of ssDNA oligonucleotides were designed as sequences complementary to the sticky ends of *Bbs*I in plasmid pEC-02 or different sites inside DRs.

**FIGURE 2:**
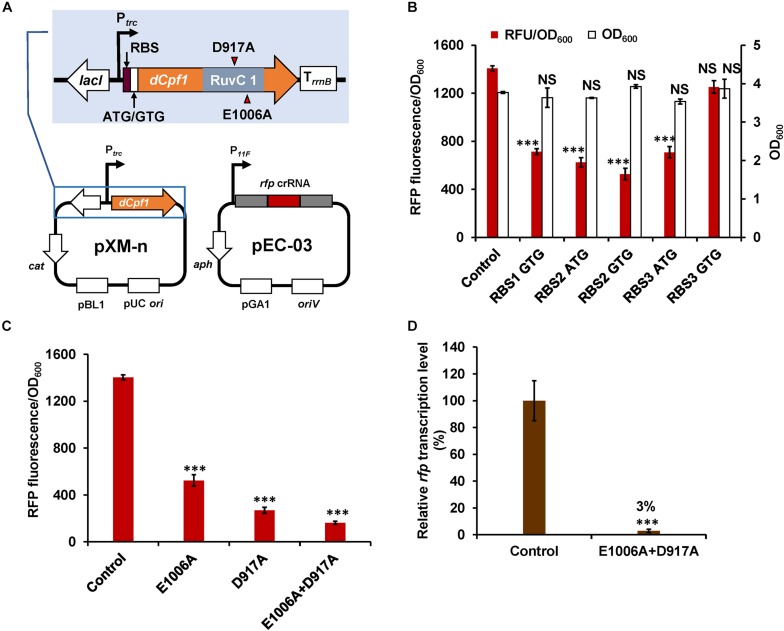
Development of CRISPR-dCpf1-mediated gene repression system in *C. glutamicum*. **(A)** Schematic diagram of the CRISPR-dCpf1 tool plasmids for *rfp* repression. **(B)** Effects of different RBSs and start codons of *dCpf1* gene on *rfp* repression efficiency and cell growth. **(C)** Effects of different dCpf1 variants on *rfp* repression efficiency. **(D)** Relative mRNA levels of *rfp* in *C. glutamicum* ATCC 13032::*rfp* strains with or without the CRISPR-dCpf1 system. The strain expressing *rfp*-targeting crRNA but no dCpf1 was used as a control. IPTG (1 mM) was added for inducing dCpf1 expression. Error bars indicate standard deviations from three parallel experiments. All *t*-tests compare the fluorescence, OD_600_ or transcription level obtained by strains expressing *rfp*-targeting crRNA and dCpf1 against control strain expressing *rfp*-targeting crRNA but no dCpf1 (^∗∗∗^*P* < 0.001; NS, non-significant).

To construct a DNase-deactivated Cpf1 variant, E1006A mutation was introduced to Cpf1 of *F. novicida* as described previously (Zetsche et al., 2015). The *dCpf1* gene was cloned to pXMJ19 plasmid under control of an IPTG-inducible promoter (P*_*trc*_*), producing pXM-01. However, transformation of pXM-01 into *C. glutamicum* failed. Previous studies reported that excess expression of dCas9 in *E. coli* inhibited cell growth and down-regulation of its expression level relieved this adverse effect (Cho et al., 2018; Cui et al., 2018). To decrease the leaky expression of dCpf1 (E1006A) and avoid potential toxicity to host cells, two RBSs with lower translation initiation efficiency were selected to replace the original strong RBS (Zhang et al., 2015). In addition, the start codon ATG of *dCpf1* was also replaced by GTG. All of the five new modified plasmids were successfully transformed into *C. glutamicum*. To construct an expression plasmid for crRNA array, a cassette consisting of a constitutive promoter (P*_11__*F*_*), two direct repeats (DRs), a *ccdB* flanked by two *Bbs*I sites, and a terminator was first assembled and cloned to pEC-XK99E, resulting the basic plasmid pEC-02. Bacterial toxin gene *ccdB* was used as a counter-selectable marker for negative selection in plasmid construction (Wang et al., 2014). By using Golden Gate assembly and annealed ssDNA oligodeoxynucleotides, multiple spacers and DRs could be efficiently assembled in a single reaction (Figure 1). To rapidly determine repression efficiency of aforementioned dCpf1 (E1006A) expression systems, RFP was used as a reporter. A spacer sequence targeting the template strand of *rfp* with a 5′-BTTV-3′ PAM sequence preferred by *F. novicida* Cpf1 (Leenay et al., 2016) was selected and inserted into plasmid pEC-02 (Figure 1). The potential off-target sites of *rfp*-targeting crRNA were examined using Cas-OFFinder (Bae et al., 2014). The resulting plasmid pEC-03 and different dCpf1 (E1006A) plasmids were co-transformed into RFP expressing *C. glutamicu*m strain ATCC 13032::*rfp* (Figure 2A). The RFP fluorescence intensities were reduced in all the strains expressing both *rfp*-targeting crRNA and dCpf1 (E1006A). The highest repression efficiency (63%) was achieved when RBS2 (AAAGGTGGTTCAT) and start codon of GTG were used for dCpf1 (E1006A) expression. No significant changes in cell growth were observed when dCpf1 was expressed with different start codons or RBSs (Figure 2B). Furthermore, individual expression of *rfp*-targeting crRNA or dCpf1 (E1006A) did not reduce RFP fluorescence intensity (Supplementary Figure S1), suggesting the observed gene repression was contributed by the CRISPR-dCpf1 system.

Either D917A or E1006A mutation in the RuvC domain of Cpf1 could completely deactivate its DNA cleavage activity (Zetsche et al., 2015). However, different mutations may affect DNA binding abilities of dCpf1 and result in different repression activities. A recent study reported that dCpf1 (D917A) showed higher repression activity than dCpf1 (E1006A) in *E. coli* (Miao et al., 2019). Therefore, another two dCpf1 variants, dCpf1 (D917A) and dCpf1 (E1006A, D917A), were constructed and evaluated for their performances in gene repression. Employment of dCpf1 (D917A) and dCpf1 (E1006A, D917A) led to 81 and 89% repression of RFP expression, respectively (Figure 2C), which is significantly higher than that obtained by dCpf1 (E1006A) (63%). qPCR further confirmed that dCpf1-based CRISPRi system significantly decreased the mRNA level of *rfp* by 97% relative to the control strain (Figure 2D). It was noticed that the repression efficiency at protein level (89%) was lower than that at mRNA level (97%). We speculate that the translation process might contribute to this difference. Similar phenomena have been observed in previous studies reporting CRISPRi (Park et al., 2018; Zhang et al., 2018). For example, Park and colleagues applied CRISPR-dCas9 to knock down *gltA* in *C. glutamicum* DM1919. The efficiency of mRNA knockdown reached 96% while the enzyme acidity was reduced by 70–80% (Park et al., 2018). Taken together, an efficient gene repression technique based on CRISPR-dCpf1 was successfully developed for *C. glutamicum* by optimizing dCpf1 expression and screening the most suitable dCpf1 variants. dCpf1 (E1006A, D917A) was used in subsequent experiments due to its higher repression activity.

### CRISPR-dCpf1-Mediated Multiplex Gene Repression in *C. glutamicum*

**FIGURE 3:**
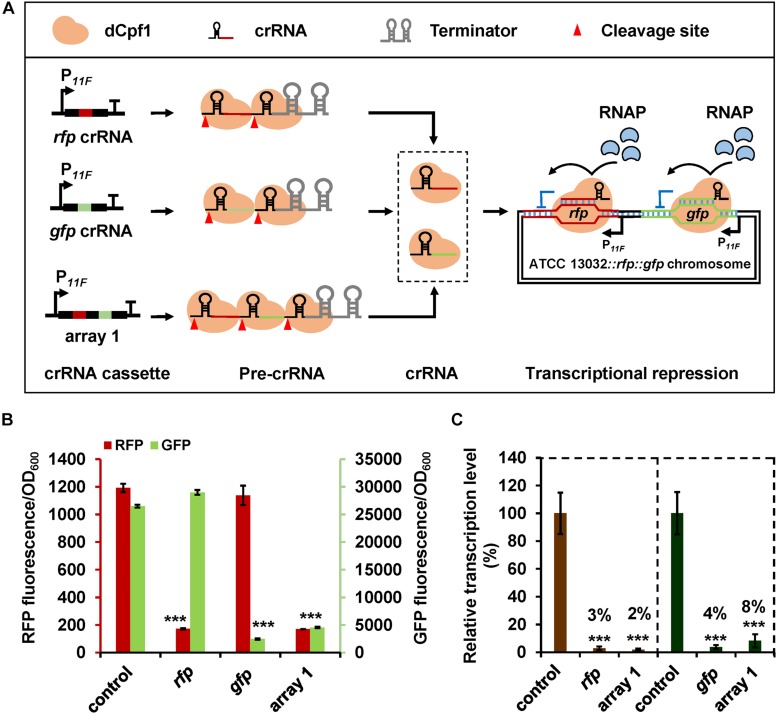
CRISPR-dCpf1-mediated multiplex gene repression in *C. glutamicum.* **(A)** Schematic diagram of application of CRISPR-dCpf1 for multiplex gene repression. Chromosomal expressed *rfp* and *gfp* were employed as reporter genes. **(B)** Fluorescence intensities of RFP and GFP controlled by the CRISPR-dCpf1 system with *rfp*-targeting crRNA, *gfp*-targeting crRNA, and crRNA array 1 harboring both *rfp*- and *gfp*-targeting spacers. The strain expressing dCpf1 but no crRNA was used as the control. **(C)** Relative transcription levels of *rfp* and *gfp* in *C. glutamicum* ATCC 13032::*rfp*::*gfp* strains expressing dCpf1 and different crRNAs. The strain expressing dCpf1 but no crRNA was used as the control. Error bars indicate standard deviations from three parallel experiments. All *t*-tests compare the fluorescence or transcription level obtained by strains expressing dCpf1 and crRNA against control strain expressing dCpf1 but no crRNA (***P < 0.001).

Next, we tested the application of CRISPR-dCpf1 system in multiple gene repression in *C. glutamicum*. A dual-fluorescence reporter system was constructed with RFP and GFP, resulting in strain ATCC 13032::*rfp*::*gfp* (Supplementary Figure S2A). RFP and GFP fluorescence can be determined in the recombinant strain without interfering with each other, which can be used for double gene repression test (Supplementary Figure S2B). A crRNA array harboring *rfp*- and *gfp*-targeting spacers (array 1) and two crRNAs with individual *rfp*- or *gfp*-targeting spacers were assembled (Figure 3A). The crRNA expression plasmid was introduced into strain 13032::*rfp*::*gfp* with plasmid pXM-07 and RFP and GFP fluorescence intensities were detected. Expression of crRNA array 1 decreased fluorescence of RFP and GFP by 86 and 83%, respectively (Figure 3B). Transcription of *rfp* and *gfp* was also decreased by 98 and 92%, respectively (Figure 3C). The efficiencies of simultaneous repression of *rfp* and *gfp* obtained with crRNA array 1 were similar to those obtained with crRNAs harboring individual *rfp*- or *gfp*-targeting spacers (∼90% at protein level and ∼96% at mRNA level) (Figures 3B,C). Furthermore, the expression of *rfp* (or *gfp*) was not significantly affected by the *gfp*-targeting (or *rfp*-targeting) crRNA (Figure 3B). The results demonstrate the high efficiency and specificity of CRISPR-dCpf1-mediated multiplex gene repression.

### Multiplex Gene Repression by CRISPR-dCpf1 for Enhancing Lysine Production

**FIGURE 4:**
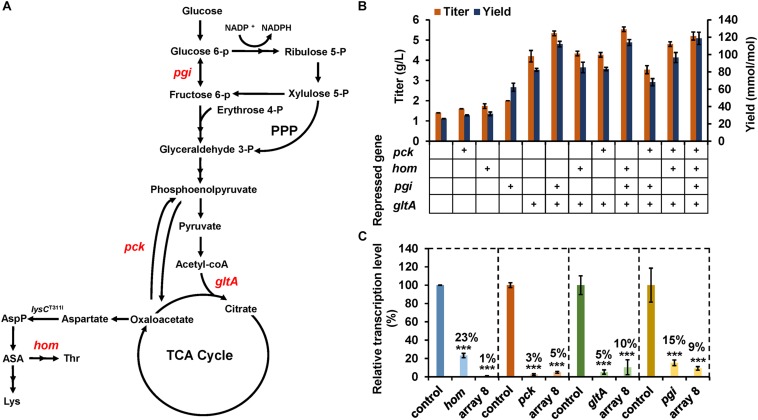
Enhancing lysine production by CRISPR-dCpf1-mediated multiplex gene repression. **(A)** Lysine biosynthesis pathway of *C. glutamicum*. Target genes repressed by CRISPR-dCpf1 are colored. AspP, aspartyl phosphate; ASA, aspartate semialdehyde; Thr, threonine; Lys, lysine. **(B)** Effects of CRISPR-dCpf1-mediated single and multiple genes repression on lysine production. SCgL30 strains co-expressing dCpf1 with an individual crRNA or a crRNA array were cultivated in fermentation medium for 24 h. An engineered strain expressing dCpf1 but no crRNA was used as the control. **(C)** Relative transcription levels of target genes. Four target genes (*gltA*, *pgi*, *hom*, and *pck*) were simultaneously repressed by dCpf1 and crRNA array 8. SCgL30 strain expressing dCpf1 but no crRNA was used as the control. Error bars indicate standard deviations from three parallel experiments. All *t*-tests compare the transcription level obtained by strains expressing dCpf1 and crRNA against control strain expressing dCpf1 but no crRNA (***P < 0.001).

To explore the potential of CRISPR-dCpf1 system for pathway engineering via endogenous gene regulation, four genes (*gltA*, *pck*, *pgi*, and *hom*) were selected as targets for enhancing lysine production in *C. glutamicum* (Figure 4A). Repression of *gltA* (encoding citrate synthase) and *pck* (encoding phosphoenolpyruvate carboxykinase) is expected to increase availability of oxaloacetate, which is the precursor for lysine biosynthesis (van Ooyen et al., 2012; Eggeling and Bott, 2015; Zhou and Zeng, 2015; Park et al., 2018). The disruption of *pgi* (encoding glucose-6-phosphate isomerase) would benefit lysine production by improving NADPH supply via enhancing pentose-phosphate pathway flux (Marx et al., 2003). Weakening threonine biosynthesis pathway branch by repressing homoserine dehydrogenase (encoded by *hom*) activity would also result in lysine accumulation due to an enhanced flux to lysine synthesis (Eggeling and Bott, 2015).

Firstly, single gene repression for these endogenous genes was studied. Four crRNAs were designed to target the 5′-end of the coding regions of target genes and cloned to pEC-02, respectively. The resultant crRNA expression plasmid was transformed into lysine-producing strain SCgL30 with plasmid pXM-07. Fermentation in 24-well plates was performed to evaluate lysine production. Compared with the control strain, repression of *pck*, *hom*, *pgi*, and *gltA* enhanced extracellular lysine concentrations by 14.3, 23.8, 42.9, and 200%, respectively (Figure 4B). The yields of glucose to lysine conversion were also increased from 25.9 to 29.8, 31.5, 62.2, and 82.3 mmol/mol by repression of *pck*, *hom*, *pgi*, and *gltA*, respectively (Figure 4B). Subsequently, mRNA levels of the target genes were analyzed. As expected, transcription of these genes was significantly down-regulated by 77–97% in the recombinant strains (Figure 4C). These results indicated that CRISPR-dCpf1 system could be employed to efficiently repress endogenous genes and used for metabolic engineering in *C. glutamicum*. The *gltA* gene was also selected as a target for improving lysine production via CRISPR-dCas9 and lysine riboswitch in previous studies and 40–60% improvements in lysine yield were obtained (Zhou and Zeng, 2015; Park et al., 2018). The differences in specific values may be mainly ascribed to the different repression efficiencies and mechanisms of these regulatory tools.

Next, combinational repression of multiple target genes was performed to investigate the best combination for lysine production. Considering the superior effect of *gltA* repression on lysine production, all the combinations contained *gltA* repression. The highest lysine titer (5.5 g/L), which was 4.0-fold higher compared with the control strain, was obtained by simultaneously repressing *gltA*, *pgi*, and *hom*. Quadruple repression of *gltA*, *pgi*, *hom*, and *pck* resulted in a slight decrease in lysine titer (5.2 g/L) but increase in lysine yield (119.0 mmol/mol) (Figure 4B), compared with the triple gene repression. Further qPCR analysis revealed that the mRNA levels of *gltA*, *pgi*, *hom*, and *pck* were simultaneously reduced by 90, 91, 99, and 95%, respectively (Figure 4C). Unexpectedly, quadruple repression of *gltA*, *pgi*, *hom*, and *pck* led to an increased repression efficiency of *hom* (99%), compared to individual repression of *hom* (77%). To investigate the possible cause, we performed a blast between the spacers of *gltA*-, *pgi*-, and *pck*-targeting crRNAs and the *hom* transcript. Two potential off-target sites with functional 5′-BTTV-3′ PAM sequences were identified for *gltA*- and *pgi*-targeting crRNA in *hom* transcript with nine mismatches (Supplementary Figure S3). It is uncertain whether the extra repression of *hom* in quadruple repression experiment was caused by such weak off-target effects or other unknown mechanism. In summary, these results indicate that the CRISPR-dCpf1 system could repress four genes simultaneously with high efficiencies and could be employed for quickly identifying the optimum gene regulation strategy for metabolic engineering in *C. glutamicum*.

## Discussion

Due to the complexity of cell metabolism, multiple metabolic engineering targets need to be manipulated to balance metabolic flux and maximize biosynthesis of desired products (Jones et al., 2015; Lee and Kim, 2015; Nielsen and Keasling, 2016). For example, genes involved in byproduct formation and intermediate competition are usually knocked out to develop efficient microbial cell factories. However, consecutive deletion of multiple genes is time consuming and laborious (Schafer et al., 1994). Furthermore, for those genes which are essential for cell growth and metabolism, gene knockout is probably not the best choice. As an alternative, multiplex gene repression can be used for optimizing metabolic pathway and is preferred for balancing cell growth and hyper-production of molecules of interests (Solomon et al., 2012; Na et al., 2013). CRISPR-dCas9- and sRNA-mediated gene repression systems have been developed and used for the metabolic engineering of *C. glutamicum*. By introducing multiple crRNA or sRNA expression cassettes, up to three genes were down-regulated simultaneously in *C. glutamicum* (Cleto et al., 2016; Zhang et al., 2016; Park et al., 2018, 2019; Gauttam et al., 2019; Sun et al., 2019). However, the complexity of assembling multiple expression cassettes and genetic instability of plasmid with repeated sequences make simultaneous repression of more target genes difficult. In this study, dCpf1-based CRISPRi system was successfully established for multiplex gene repression in *C. glutamicum*. Due to its inherent function of maturing crRNA, a single crRNA array is sufficient for repressing multiple target genes by CRISPR-dCpf1, which can largely simplify plasmid construction. We tested repression of four genes by the CRISPR-dCpf1 system with a crRNA array, which resulted in over 90% repression efficiencies for all targets. We envision that the CRISPR-dCpf1 system can facilitate simultaneous repression of more target genes in *C. glutamicum*.

CRISPR-dCpf1 has been applied in *E. coli* and *Streptomyces* to modulate multiple genes expression. In *E. coli* and our *C. glutamicum* cases, quadruple genes repression showed similar efficiencies with individual gene repression (Zhang et al., 2017), while increasing the number of target genes in *Streptomyces* decreased repression efficiency (∼70% for triple genes vs. 82.1–95.2% for single gene) (Li et al., 2018). Another interesting phenomenon is dCpf1 (D917A) outperformed dCpf1 (E1006A) and dCpf1 (E1006A, D917A) in *E. coli* (Miao et al., 2019). In *C. glutamicum*, however, dCpf1 (E1006A, D917A) showed the highest repression activity compared to the rest two variants with a single mutation. These results suggest that the repression activity of dCpf1 might be host-dependent. For practical applications, target genes may need to be repressed at various levels. To this end, several strategies are applicable. It has been reported that the repression efficiency of CRISPR-dCpf1 system can be adjusted by using truncated or mutated spacers, engineered DRs, and difference PAM patterns (Liu et al., 2019; Miao et al., 2019; Wang et al., 2019). Tuning the expression levels of CRISPRi components (dCpf1 and crRNA) with various constitutive or inducible promoters can also facilitate a quantitative control of the repression efficiency (Fontana et al., 2018). Similar strategies can be investigated in *C. glutamicum* to achieve gradient knockdown of multiple targets in further studies.

## Data Availability Statement

All data generated or analyzed during this study are included in this published article and Supplementary Material. The authors are willing to provide any additional data and materials related to this research that may be requested for research purposes.

## Author Contributions

ML, JC, PZ, and JS conceptualized the project and designed the study. ML, JC, and JH conducted the experiments and collected data. ML, JC, YW, and JL performed data analysis. ML, JC, YW, PZ, and JS composed the manuscript. PZ, JS, and NC provided critical feedback on the manuscript and provided resources. All authors read and approved the manuscript.

## Conflict of Interest

The authors declare that the research was conducted in the absence of any commercial or financial relationships that could be construed as a potential conflict of interest.
