# Supplementary material for: Efficient Multiplex Gene Repression by CRISPR-dCpf1 in Corynebacterium glutamicum
Source: Front Bioeng Biotechnol. 2020 Apr 24;8:357. doi: 10.3389/fbioe.2020.00357 (PMC7193084; doi:10.3389/fbioe.2020.00357)
Supplement: Supplementary file 1 [file Table_1.DOCX]

Supplementary Material


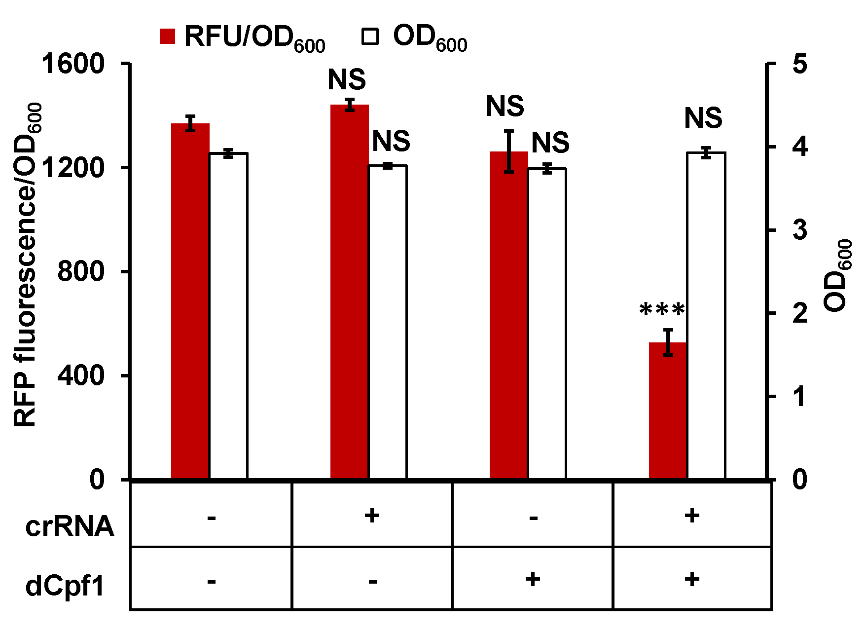


**Figure S1.** Fluorescence intensity of RFP controlled by the CRISPR-dCpf1 system. Individual expression of dCpf1(E1006A, RBS2 GTG) or *rfp*-targeting crRNA did not reduce RFP fluorescence intensity in ATCC 13032::*rfp*. IPTG (1 mM) was used for inducing dCpf1 expression. Error bars indicate standard deviations from three parallel experiments. ALL *t*-tests compare the fluorescence or OD_600_ obtained by strains expressing *rfp*-targeting crRNA and/or dCpf1 against control strain without *rfp*-targeting crRNA and dCpf1 (****P*<0.001; NS, non-significant).


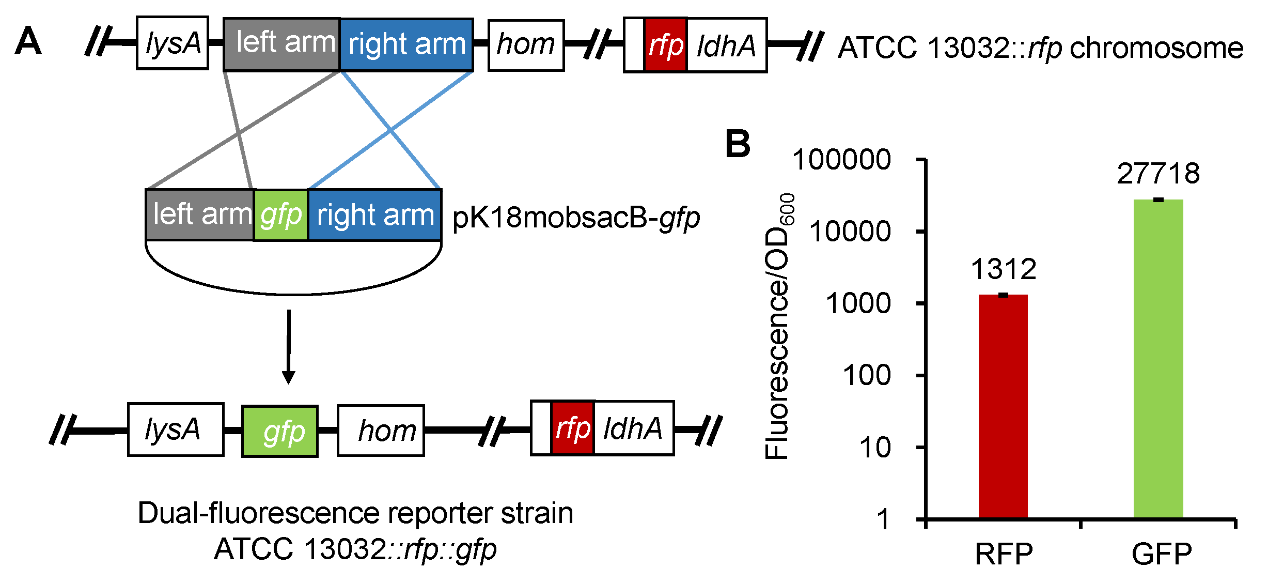


**Figure S2.** Construction of a dual-fluorescence reporter strain ATCC 13032::*rfp*::*gfp* **(A)** and determination of its RFP and GFP fluorescence intensities **(B)**. A previously constructed *rfp*-expressing strain ATCC 13032::*rfp* was used to construct the dual-fluorescence reporter strain ATCC 13032::*rfp*::*gfp*, which constitutively expresses *rfp* and *gfp*. Plasmid pK18*mobsacB*-*gfp* was used to integrate a *gfp* expression cassette downstream *lysA* gene via homologous recombination. Error bars indicate standard deviations from three parallel experiments.


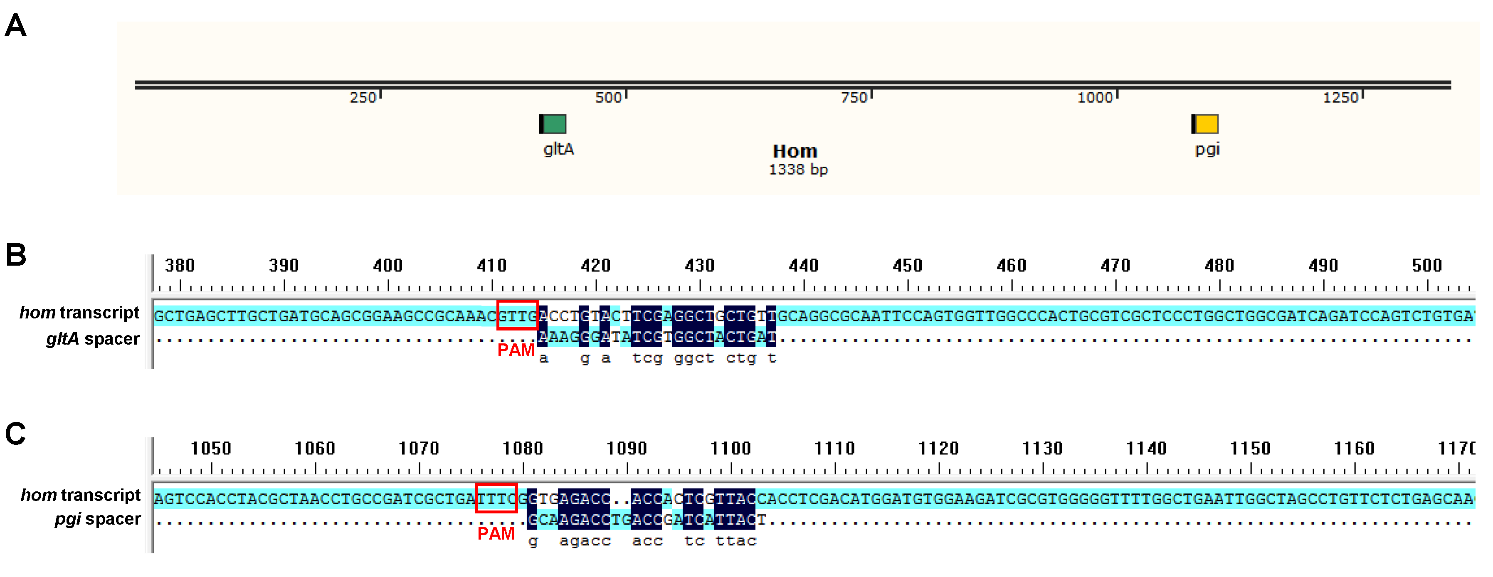


**Figure S3.** Potential off-target analysis of *gltA*- and *pgi*-targeting crRNAs in *hom* transcript. **(A)** Sites of potential pairing between *gltA*- and *pgi*-targeting crRNAs and *hom* transcript. **(B)** Sequence similarity between the spacer of *gltA*-targeting crRNA and *hom* transcript. **(C)** Sequence similarity between the spacer of *pgi*-targeting crRNA and *hom* transcript. PAM sequence is highlighted in red.

**Table S1** Bacterial strains and plasmids used in this study

| **Strain or Plasmid** | **Relevant characteristic^a^** | **Source** |
| --- | --- | --- |
| **Strains** |  |  |
| *E. coli* |  |  |
| DH5α | Host for cloning | Lab stock |
| Trans DB.3.1 | Host for cloning of plasmid harboring *ccdB* | Lab stock |
| *C. glutamicum* |  |  |
| ATCC 13032 | Wild-type strain | ATCC |
| ATCC 13032::*rfp* | ATCC 13032 derivative with chromosomal insertion of a *rfp* expression cassette | ([Liu et al., 2017](#_ENREF_3)) |
| SCgL30 | ATCC 13032 derivative with T311I mutation of LysC | Lab stock |
| ATCC 13032::*rfp*::*gfp* | ATCC 13032::*rfp* derivative with chromosomal insertion of a *gfp* expression cassette | This study |
| **Plasmids** |  |  |
| pXMJ19 | Expression vector of *C. glutamicum*, IPTG-inducible promoter P*_tac_*, Cm^R^ | ([Jakoby et al., 1999](#_ENREF_1)) |
| pEC-XK99E | Expression vector of *C. glutamicum*, IPTG-inducible promoter P*_trc_*, Km^R^ | ([Kirchner and Tauch, 2003](#_ENREF_2)) |
| pK18*mobsacB* | Integration vector of *C. glutamicum*, Km^R^ | ([Schafer et al., 1994](#_ENREF_4)) |
| pXM-*gfp* | Source of *gfp* gene | ([Sun et al., 2019](#_ENREF_5)) |
| pgRNA-*ccdB* | Source of *ccdB* gene | ([Wang et al., 2018](#_ENREF_6)) |
| pY003 | Source of *FnCpf1* gene, Cm^R^ | ([Zetsche et al., 2015](#_ENREF_7)) |
| pY003-*dCpf1* | pY003 derivative carrying dCpf1(E1006A) encoding gene | This study |
| pK18*mobsacB*-*gfp* | pK18*mobsacB* derivative carrying *gfp* expression cassette for chromosomal integration | This study |
| pXM-01 | pXMJ19 derivative carrying dCpf1(E1006A) encoding gene | This study |
| pXM-02 | pXM-01 derivative, the start codon of *dCpf1* replaced with GTG | This study |
| pXM-03 | pXM-01 derivative, RBS-1 of *dCpf1* replaced with RBS-2 | This study |
| pXM-04 | pXM-02 derivative, RBS-1 of *dCpf1* replaced with RBS-2 | This study |
| pXM-05 | pXM-01 derivative, RBS-1 of *dCpf1* replaced with RBS-3 | This study |
| pXM-06 | pXM-02 derivative, RBS-1 of *dCpf1* replaced with RBS-3 | This study |
| pXM-07 | pXM-04 derivative carrying dCpf1(E1006A, D917A) encoding gene | This study |
| pXM-08 | pXM-07 derivative carrying dCpf1(D917A) encoding gene | This study |
| pEC-01 | pEC-XK99E derivative, P*_trc_* replaced with P*_11F_* | This study |
| pEC-02 | pEC-01 derivative carrying the *ccdB* gene | This study |
| pEC-03 | pEC-02 derivative carrying the *rfp* crRNA | This study |
| pEC-08 | pEC-02 derivative carrying the *gfp* crRNA | This study |
| pEC-09 | pEC-02 derivative carrying the crRNA array 1, targeting *rfp* and *gfp* genes | This study |
| pEC-10 | pEC-02 derivative carrying the *gltA* crRNA | This study |
| pEC-11 | pEC-02 derivative carrying the *hom* crRNA | This study |
| pEC-12 | pEC-02 derivative carrying the *pck* crRNA | This study |
| pEC-13 | pEC-02 derivative carrying the *pgi* crRNA | This study |
| pEC-14 | pEC-02 derivative carrying the crRNA array 2, targeting *gltA* and *hom* genes | This study |
| pEC-15 | pEC-02 derivative carrying the crRNA array 3, targeting *gltA* and *pgi* genes | This study |
| pEC-16 | pEC-02 derivative carrying the crRNA array 4, targeting *gltA* and *pck* genes | This study |
| pEC-17 | pEC-02 derivative carrying the crRNA array 5, targeting *gltA*, *pgi* and *hom* genes | This study |
| pEC-18 | pEC-02 derivative carrying the crRNA array 6, targeting *gltA*, *pgi* and *pck* genes | This study |
| pEC-19 | pEC-02 derivative carrying the crRNA array 7, targeting *gltA*, *hom* and *pck* genes | This study |
| pEC-20 | pEC-02 derivative carrying the crRNA array 8, targeting *gltA*, *pgi*, *hom* and *pck* genes | This study |

^a^Km^R^ and Cm^R^ represent resistance to kanamycin and chloramphenicol, respectively.

**Table S2** Primers used in this study

| **Primer** | **Sequence (5’-3’)** | **Relevance** |
| --- | --- | --- |
| pY003-E1006A-F | CGGATTTAAATTTTGGATTTAAAAGAGGGCG | pXM-01 construction |
| pY003-E1006A-R | CAAAAACCACAATAGCATTATACTC |  |
| pXM-01-F | AACTGCAGAAAGGAGTTGAGAATGTCAATTTATCAAGAA |  |
| pXM-01-R | CGGGATCCTTAGTTATTCCTATTCTGCACG |  |
| pXM-02-GTG-F | GAAAGGAGTTGAGAGTGTCAATTTATCAAGAATTTGA | pXM-02 construction |
| pXM-02-GTG-R | TTGATAAATTGACACTCTCAACTCCTTTCTGCAGGC |  |
| pXM-03-ATG-F | CAGGAAACAGAATTAATTAAGCTTAAAGGTGGTTCATATGTCAATTTATCAAGAA | pXM-03 construction |
| pXM-SD-R | TTCGAGCTCGGTACCCGGGGATCCTTAGTTATTCCTATTCTGCACGAACTC |  |
| pXM-04-GTG-F | CAGGAAACAGAATTAATTAAGCTTAAAGGTGGTTCATGTGTCAATTTATCAAGAA | pXM-04 construction |
| pXM-05-ATG-F | CAGGAAACAGAATTAATTAAGCTTAAAGGCACCCGATATGTCAATTTATCAAGAA | pXM-05 construction |
| pXM-06-GTG-F | CAGGAAACAGAATTAATTAAGCTTAAAGGCACCCGATGTGTCAATTTATCAAGAA | pXM-06 construction |
| pXM-07-F | ATATTAAGTATAGCTAGAGGTGAAAGACATTTAGC | pXM-07 construction |
| pXM-07-R | TCTTTCACCTCTAGCTATACTTAATATATGAACATC |  |
| pXM-08-F | GTGGTTTTTGAGGATTTAAATTTTGGATTTAAAAGAGGG | pXM-08 construction |
| pXM-08-R | ATTTAAATCCTCAAAAACCACAATAGCATTATACTC |  |
| pEC-01-*11F*-F | GCGACGCAACAGGTACAGTGTAATTCAAAACAGACCATGGAATTCGAG | pEC-01 construction |
| pEC-01-*11F*-R | AACATTGCCAGCTTATGTGGAGAAAAACACCACCCTGAATTGACTCTC |  |
| pEcrRNA-F | AGATAAAGGGATATCGTGGCTACTGATGAATTTCTACTGTTGTAGATACCCCGGC | pEC-02 construction |
| pEcrRNA-R | CCTTGCCGGGGTATCTACAACAGTAGAAATTCATCAGTAGCCACGATATCCCTTT |  |
| pEC-02-*ccdB*-F | GTACAGTGTAATTCAGAATTTCTACTGTTGTAGATTTGTCTTCTGGTGTCTGAGACCACGCGTGGATCC |  |
| pEC-02-*ccdB*-R | TGAGACCTTATATTCCCCAGAACATCAG |  |
| *gfp*-up-F | AATTCGAGCTCGGTACCCGGGGATCCCAGGCAATGTGCACTACGCATC | pK18*mobsacB-gfp* construction |
| *gfp*-up-R | AAGTTCGCAAAGAAACCCAGAAACC |  |
| *gfp*-down-F | GTCGAGGCTTCAGAGGTTTTATTGC |  |
| *gfp*-down-R | TTGCATGCCTGCAGGTCGACTCTAGAGTTAAAGCTTGGGGCAGATGCTG |  |
| pK18mobsacB-F | TCTAGAGTCGACCTGCAGGCATGCAA |  |
| pK18mobsacB-R | GGATCCCCGGGTACCGAGCTCGAATT |  |
| *gfp*-F | CTGGGTTTCTTTGCGAACTTTTTTCTCCACATAAGCTGGCAATG |  |
| *gfp*-R | AAAACCTCTGAAGCCTCGACTTATTTGTATAGTTCATCCATGCCATGTG |  |
| *16sRNA*-F | CGGATCGGCGTAGAGATAC | qPCR |
| *16sRNA*-R | GCTGGCAACATAAGACAAGG |  |
| *gfp*-F | GCGTTCAACTAGCAGACCATTATC |  |
| *gfp*-R | GTTCATCCATGCCATGTGTAATCC |  |
| *rfp*-F | CGAAGGTGAAGGTGAAGG |  |
| *rfp*-R | GGAACCGTACTGGAACTG |  |
| *gltA*-F | AAGGTCAAGAACAAGGAAG |  |
| *gltA*-R | AAGTAATCATCAGCCAGTG |  |
| *pgi*-F | CCTCAACAACACCGAAGAC |  |
| *pgi*-R | GGACATCAGCAGCAACATC |  |
| *pck*-F | ACATCGCAGCAGCATTCC |  |
| *pck*-R | CGTCGCCAACAACCTGAG |  |
| *hom*-F | AGTCCACCTACGCTAACC |  |
| *hom*-R | ATTCCTTGCTCAGAGAACAG |  |

**Table S3** crRNAs and gene expression elements used in this study

| **Gene element** | **Sequence (5’-3’)** |
| --- | --- |
| **crRNA** |  |
| *rfp* | GAATTTCTACTGTTGTAGATaaagttcgtatggaaggttccgttGAATTTCTACTGTTGTAGAT |
| *gfp* | GAATTTCTACTGTTGTAGATcactactggaaaactacctgttcGAATTTCTACTGTTGTAGAT |
| *gltA* | GAATTTCTACTGTTGTAGATaaagggatatcgtggctactgatGAATTTCTACTGTTGTAGAT |
| *hom* | GAATTTCTACTGTTGTAGATaccccggcaagggtcccggctcaGAATTTCTACTGTTGTAGAT |
| *pgi* | GAATTTCTACTGTTGTAGATgcaagacctgaccgatcattactGAATTTCTACTGTTGTAGAT |
| *pck* | GAATTTCTACTGTTGTAGATtgttcgttgatggatcccaggctGAATTTCTACTGTTGTAGAT |
| array 1 | GAATTTCTACTGTTGTAGATaaagttcgtatggaaggttccgttGAATTTCTACTGTTGTAGATcactactggaaaactacctgttcGAATTTCTACTGTTGTAGAT |
| array 2 | GAATTTCTACTGTTGTAGATaaagggatatcgtggctactgatGAATTTCTACTGTTGTAGATaccccggcaagggtcccggctcaGAATTTCTACTGTTGTAGAT |
| array 3 | GAATTTCTACTGTTGTAGATaaagggatatcgtggctactgatGAATTTCTACTGTTGTAGATgcaagacctgaccgatcattactGAATTTCTACTGTTGTAGAT |
| array 4 | GAATTTCTACTGTTGTAGATaaagggatatcgtggctactgatGAATTTCTACTGTTGTAGATtgttcgttgatggatcccaggctGAATTTCTACTGTTGTAGAT |
| array 5 | GAATTTCTACTGTTGTAGATaaagggatatcgtggctactgatGAATTTCTACTGTTGTAGATgcaagacctgaccgatcattactGAATTTCTACTGTTGTAGATaccccggcaagggtcccggctcaGAATTTCTACTGTTGTAGAT |
| array 6 | GAATTTCTACTGTTGTAGATaaagggatatcgtggctactgatGAATTTCTACTGTTGTAGATgcaagacctgaccgatcattactGAATTTCTACTGTTGTAGATtgttcgttgatggatcccaggctGAATTTCTACTGTTGTAGAT |
| array 7 | GAATTTCTACTGTTGTAGATaaagggatatcgtggctactgatGAATTTCTACTGTTGTAGATaccccggcaagggtcccggctcaGAATTTCTACTGTTGTAGATtgttcgttgatggatcccaggctGAATTTCTACTGTTGTAGAT |
| array 8 | GAATTTCTACTGTTGTAGATaaagggatatcgtggctactgatGAATTTCTACTGTTGTAGATgcaagacctgaccgatcattactGAATTTCTACTGTTGTAGATaccccggcaagggtcccggctcaGAATTTCTACTGTTGTAGATtgttcgttgatggatcccaggctGAATTTCTACTGTTGTAGAT |
| **Promoter** |  |
| P*_trc_* | TTGACAATTAATCATCGGCTCGTATAATGTGTGGAATTGTGAGCGGATAACAATTTC |
| P*_11F_* | TTTTCTCCACATAAGCTGGCAATGTTGCGACGCAACAGGTACAGTGTAATTCA |
| **RBS** |  |
| RBS 1 | AAAGGAGTTGAGA |
| RBS 2 | AAAGGTGGTTCAT |
| RBS 3 | AAAGGCACCCGAT |
| **Terminator** |  |
| T1/T2 | ATAAAACGAAAGGCTCAGTCGAAAGACTGGGCCTTTCGTTTTATCTGTTGTTTGTCGGTGAACGCTCTCCTGAGTAGGACAAATCCGCCGGGAGCGGATTTGAACGTTGCGAAGCAACGGCCCGGAGGGTGGCGGGCAGGACGCCCGCCATAAACTGCCAGGCATCAAATTAAGCAGAAGGCCATCCTGACGGATGGCCTTTTTGCGTTTCTACAAACTCTT |

^a^Direct repeat and spacer sequences of crRNAs are indicated as capital and lowercase letters, respectively.

**Table S4** ssDNA oligonucleotides used in this study

| **Oligonucleotide** | **Sequence (5’-3’)** | **Relevance** |
| --- | --- | --- |
| *ccdB*-*rfp*-F | AGATAAAGTTCGTATGGAAGGTTCCGTT | pEC-03 construction |
| *ccdB*-*rfp*-R | ATTCAACGGAACCTTCCATACGAACTTT |  |
| *ccdB-gfp*-F | AGATAGAATGAGTAAAGGAGAAGAACT | pEC-08 construction |
| *ccdB*-*gfp*-R | ATTCAGTTCTTCTCCTTTACTCATTCT |  |
| array1-F1 | AGATAAAGTTCGTATGGAAGGTTCCGTTGAATTTCTACT | pEC-09 construction |
| array1-R1 | CAACAGTAGAAATTCAACGGAACCTTCCATACGAACTTT |  |
| array1-F2 | GTTGTAGATAGAATGAGTAAAGGAGAAGAACT |  |
| array1-R2 | ATTCAGTTCTTCTCCTTTACTCATTCTATCTA |  |
| *ccdB*-*gltA*-F | AGATAAAGGGATATCGTGGCTACTGAT | pEC-10 construction |
| *ccdB*-*gltA*-R | ATTCATCAGTAGCCACGATATCCCTTT |  |
| *ccdB*-*hom*-F | AGATACCCCGGCAAGGGTCCCGGCTCA | pEC-11 construction |
| *ccdB*-*hom*-R | ATTCTGAGCCGGGACCCTTGCCGGGGT |  |
| *ccdB*-*pck*-F | AGATTGTTCGTTGATGGATCCCAGGCT | pEC-12 construction |
| *ccdB*-*pck*-R | ATTCAGCCTGGGATCCATCAACGAACA |  |
| *ccdB*-*pgi*-F | AGATGCAAGACCTGACCGATCATTACT | pEC-13 construction |
| *ccd B*-*pgi*-R | ATTCAGTAATGATCGGTCAGGTCTTGC |  |
| array2-8-F1 | AGATAAAGGGATATCGTGGCTACTGATGAATTTCTACT | pEC-14-pEC-20 construction |
| array2-8-R1 | CAACAGTAGAAATTCATCAGTAGCCACGATATCCCTTT |  |
| array2-F2 | GTTGTAGATACCCCGGCAAGGGTCCCGGCTCA | pEC-14 construction |
| array2-R2 | ATTCTGAGCCGGGACCCTTGCCGGGGTATCTA |  |
| array3-F2 | GTTGTAGATGCAAGACCTGACCGATCATTACT | pEC-15 construction |
| array3-R2 | ATTCAGTAATGATCGGTCAGGTCTTGCATCTA |  |
| array4-F2 | GTTGTAGATTGTTCGTTGATGGATCCCAGGCT | pEC-16 construction |
| array4-R2 | ATTCAGCCTGGGATCCATCAACGAACAATCTA |  |
| array5/6/8-F2 | GTTGTAGATGCAAGACCTGACCGATCATTACTGAATTTC | pEC-17, pEC-18, pEC-20 construction |
| array5/6/8-R2 | AGTAGAAATTCAGTAATGATCGGTCAGGTCTTGCATCTA |  |
| array5-F3 | TACTGTTGTAGATACCCCGGCAAGGGTCCCGGCTCA | pEC-17 construction |
| array5-R3 | ATTCTGAGCCGGGACCCTTGCCGGGGTATCTACAAC |  |
| array6-F3 | TACTGTTGTAGATTGTTCGTTGATGGATCCCAGGCT | pEC-18 construction |
| array6-R3 | ATTCAGCCTGGGATCCATCAACGAACAATCTACAAC |  |
| array7-F2 | GTTGTAGATACCCCGGCAAGGGTCCCGGCTCAGAATTTC | pEC-19 construction |
| array7-R2 | AGTAGAAATTCTGAGCCGGGACCCTTGCCGGGGTATCTA |  |
| array7-F3 | TACTGTTGTAGATTGTTCGTTGATGGATCCCAGGCT |  |
| array7-R3 | ATTCAGCCTGGGATCCATCAACGAACAATCTACAAC |  |
| array8-F3 | TACTGTTGTAGATACCCCGGCAAGGGTCCCGGCTCAGAATTTCT | pEC-20 construction |
| array8-R3 | CAGTAGAAATTCTGAGCCGGGACCCTTGCCGGGGTATCTACAAC |  |
| array8-F4 | ACTGTTGTAGATTGTTCGTTGATGGATCCCAGGCT |  |
| array8-R4 | ATTCAGCCTGGGATCCATCAACGAACAATCTACAA |  |

**References**

Jakoby, M., Ngouoto-Nkili, C.E., and Burkovski, A. (1999). Construction and application of new *Corynebacterium glutamicum* vectors. *Biotechnol. Tech.* 13, 437–441. doi: Doi 10.1023/A:1008968419217

Kirchner, O., and Tauch, A. (2003). Tools for genetic engineering in the amino acid-producing bacterium *Corynebacterium glutamicum*. *J. Biotechnol.* 104, 287–299. doi: 10.1016/S0168-1656(03)00148-2

Liu, J., Wang, Y., Lu, Y.J., Zheng, P., Sun, J.B., and Ma, Y.H. (2017). Development of a CRISPR/Cas9 genome editing toolbox for *Corynebacterium glutamicum*. *Microb. Cell Fact.* 16, 205. doi: 10.1186/s12934-017-0815-5

Schafer, A., Tauch, A., Jager, W., Kalinowski, J., Thierbach, G., and Puhler, A. (1994). Small mobilizable multi-purpose cloning vectors derived from the *Escherichia coli* plasmids pK18 and pK19: selection of defined deletions in the chromosome of *Corynebacterium glutamicum*. *Gene* 145, 69–73. doi: 10.1016/0378-1119(94)90324-7

Sun, D.H., Chen, J.Z., Wang, Y., Li, M.Y., Rao, D.M., Guo, Y.M., et al. (2019). Metabolic engineering of *Corynebacterium glutamicum* by synthetic small regulatory RNAs. *J. Ind. Microbiol. Biotechnol.* 46, 203–208. doi: 10.1007/s10295-018-02128-4

Wang, Y., Liu, Y., Liu, J., Guo, Y.M., Fan, L.W., Ni, X.M., et al. (2018). MACBETH: Multiplex automated *Corynebacterium glutamicum* base editing method. *Metab. Eng.* 47, 200–210. doi: 10.1016/j.ymben.2018.02.016

Zetsche, B., Gootenberg, J.S., Abudayyeh, O.O., Slaymaker, I.M., Makarova, K.S., Essletzbichler, P., et al. (2015). Cpf1 is a single RNA-guided endonuclease of a Class 2 CRISPR-Cas system. *Cell* 163, 759–771. doi: 10.1016/j.cell.2015.09.038
